# Assessment of Quality of Life of Chronic Myeloid Leukemia Patients by Using the EORTC QLQ-C30

**DOI:** 10.4274/tjh.2016.0409

**Published:** 2017-06-01

**Authors:** Mehmet Can Uğur, Yaşar Bekir Kutbay, Özge Özer Kaya, Cengiz Ceylan

**Affiliations:** 1 Tepecik Training and Research Hospital, Clinic of Internal Medicine, İzmir, Turkey; 2 Tepecik Training and Research Hospital, Genetic Diagnostic Center, İzmir, Turkey; 3 Tepecik Training and Research Hospital, Clinic of Hematology, İzmir, Turkey

**Keywords:** Cytogenetic, Chronic myeloid leukemia, Molecular hematology, Life-quality, Dasatinib, Nilotinib

## To The Editor,

Depression is determined in 15%-25% of patients with cancer and it is accepted as a comorbid problem with poor prognosis. The quality of life of these patients is determined to be poor [1,2]. We aimed to study the quality of life of patients using new forms of imatinib, dasatinib, or nilotinib.

We analyzed 56 chronic myeloid leukemia patients followed in the İzmir Tepecik Training and Research Hospital Department of Hematology. Patients were followed from 2005 to 2015. We included patients who were >18 years of age, BCR-ABL-positive based on polymerase chain reaction results, using first- or second-generation tyrosine kinase inhibitors (TKIs) in the last 6 months, and in the chronic phase of the disease.

The Turkish version of the European Organisation for Research and Treatment of Cancer Quality of Life Questionnaire-C30 (EORTC QLQ-C30) [3], the Turkish version of the Hospital Anxiety and Depression Scale [4], and the General Health Questionnaire [5] were administered to patients one-on-one. The study received approval from the ethics committee.

The demographic data and laboratory values are provided in Table 1.

In our study, we found no statistical significance between first- and second-generation TKIs. We also compared dasatinib and nilotinib as subgroups of the second generation and we found statistical significance for dasatinib against nilotinib for general life quality, emotional and cognitive functions, and fatigue parameters.

## Figures and Tables

**Table 1:**
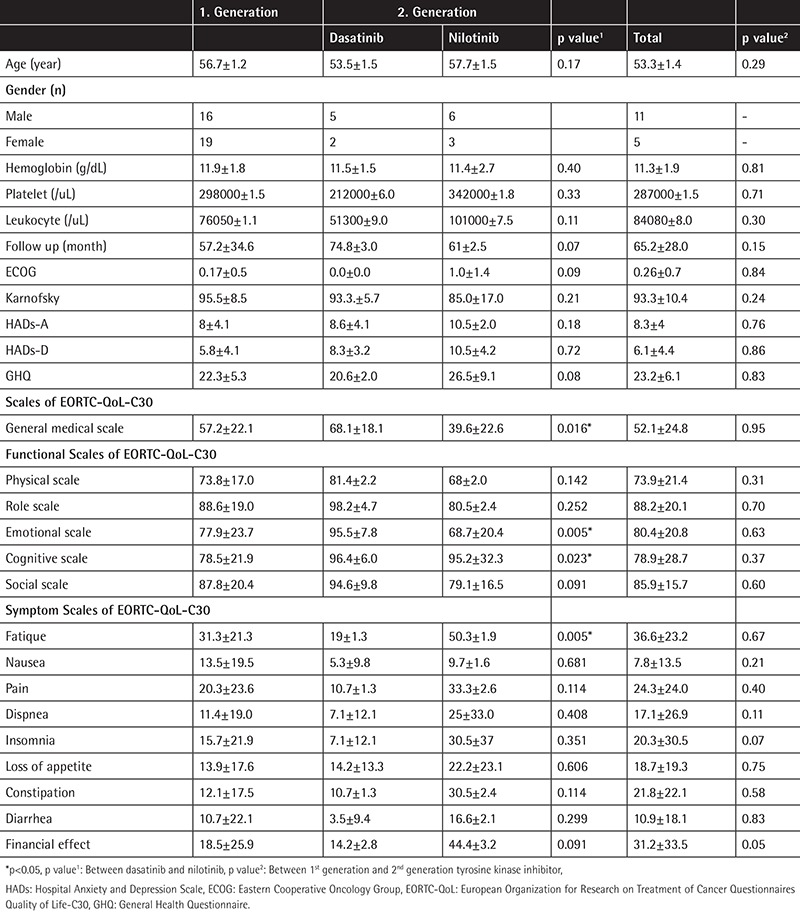
Demographic datas, laboratory findings, follow up, Hospital Anxiety and Depression Scale, General Health Questionnaire, Eastern Cooperative Oncology Group and Karnofsky scores, general medical, functional and symptom scales of European Organization for Research on Treatment of Cancer Questionnaires Quality of Life-C30 between 1^s^t generation tyrosine kinase inhibitor and 2^nd^ generation tyrosine kinase inhibitor (dasatinib and nilotinib).

## References

[1] Wilson KG, Chochinov HM, Shirko MG, Allard P, Chary S, Gagnon PR, Macmillan K, De Luca M, O’Shea F, Kuhl D, Fainsinger RL, Clinch JJ (2007). Depression and anxiety disorders in palliative cancer care. J Pain Symptom Manage.

[2] Lloyd-Williams M, Friedman T (2001). Depression in palliative care patients-a prospective study. Eur J Cancer Care (Engl).

[3] Guzelant A, Goksel T, Ozkok S, Tasbakan S, Aysan T, Bottomley A (2004). The European Organization for Research and Treatment of Cancer QLQ-C30: an examination into the cultural validity and reliability of the Turkish version of the EORTC QLQC30. Eur J Cancer Care Engl.

[4] Aydemir O (1997). Validity and reliability of Turkish version of Hospital Anxiety and Depression scale. Turkish Journal of Psychiatry.

[5] Kilic C (1996). General health questionnaire: a validity and reliability study. Turkish Journal of Psychiatry.

